# Portfolio Optimization for Binary Options Based on Relative Entropy

**DOI:** 10.3390/e22070752

**Published:** 2020-07-09

**Authors:** Peter Joseph Mercurio, Yuehua Wu, Hong Xie

**Affiliations:** 1 Department of Mathematics and Statistics, York University, Toronto, ON M3J 1P3, Canada; 2 Manulife Financial Corp., Toronto, ON M4W 1E5, Canada; hong_xie@manulife.com

**Keywords:** relative entropy, Kullback–Leibler divergence, portfolio optimization, portfolio selection, Kelly criterion, exotic options, binary options, digital options, fixed-return options, sports betting

## Abstract

The portfolio optimization problem generally refers to creating an investment portfolio or asset allocation that achieves an optimal balance of expected risk and return. These portfolio returns are traditionally assumed to be continuous random variables. In *An Entropy-Based Approach to Portfolio Optimization*, we introduced a novel non-parametric optimization method based on Shannon entropy, called return-entropy portfolio optimization (REPO), which offers a simple and fast optimization algorithm for assets with continuous returns. Here, in this paper, we would like to extend the REPO approach to the optimization problem for assets with discrete distributed returns, such as those from a Bernoulli distribution like binary options. Under a discrete probability distribution, portfolios of binary options can be viewed as repeated short-term investments with an optimal buy/sell strategy or general betting strategy. Upon the outcome of each contract, the portfolio incurs a profit (success) or loss (failure). This is similar to a series of gambling wagers. Portfolio selection under this setting can be formulated as a new optimization problem called discrete entropic portfolio optimization (DEPO). DEPO creates optimal portfolios for discrete return assets based on expected growth rate and relative entropy. We show how a portfolio of binary options provides an ideal general setting for this kind of portfolio selection. As an example we apply DEPO to a portfolio of short-term foreign exchange currency pair binary options from the NADEX exchange platform and show how it outperforms leading Kelly criterion strategies. We also provide an additional example of a gambling application using a portfolio of sports bets over the course of an NFL season and present the advantages of DEPO over competing Kelly criterion strategies.

## 1. Introduction

In our previous paper (Mercurio et al. [1]), a new class of portfolio optimization problems was introduced called return-entropy portfolio optimization (REPO). REPO uses Shannon entropy (Shannon, 1948) [2,3] as the discriminatory risk measure for portfolio selection for assets with continuous returns, as opposed to variance used by Markowitz [4] in mean-variance portfolio optimization (MVPO). REPO holds several advantages over the traditional MVPO method: REPO is robust, non-parametric, and indifferent to non-normality and asymmetry, making it an ideal approach to the traditional portfolio selection problem.

The focus of this paper is on use of the entropic portfolio optimization to select optimal portfolios of assets with discrete returns, where traditional risk management methods are not applicable. In particular, we concentrate on portfolios of binary options. Binary options have fixed discrete distributed returns of +100% or −100% as opposed to traditional continuous returns on the real line. Other instruments that behave similarly to binary options are digital options, fixed-return options (FROs), or even sports bets. These types of discrete return instruments can be assumed to follow a Bernoulli distribution with an expected probability of success and fixed profit and loss amounts. Both kinds of investment portfolios can have risk–return choices. Under a discrete probability distribution, a portfolio of binary options can be viewed as repeated short-term investments. Upon the outcome of each event, the portfolio incurs a profit or loss from the success of failure of that event. While the return for an equity portfolio is measured by the expected return of the portfolio, for a binary option portfolio we are most interested in the expected growth rate of the portfolio, as if the bet were to be repeatedly placed ad infinitum, as shown by Kelly (1956) [5].

For the risk management of binary option portfolios, we look to the concept of relative entropy, also known as Kullback–Leibler divergence (1951,1959) [6,7]. Relative entropy measures the distance between discrete distributions, and the target distribution for a portfolio of binary wagers is the uniform distribution. A binary option portfolio with only one asset (maximum relative entropy) has the highest risk, since the portfolio value will swing 100% in either direction. Allocating that same capital to an increasingly large number of binary events reduces the portfolio risk, since the expected net return will approach zero as *n* increases (minimum relative entropy). For this reason, the uniform distribution is the minimum risk portfolio for binary assets. Therefore, the risk of a binary option portfolio is quantified by its relative entropy with respect to the uniform distribution. Discrete entropic portfolio optimization (DEPO) finds the optimal allocation of capital across a series of potential binary investments in order to maximize the expected portfolio growth rate and minimize the portfolio relative entropy.

Future implications of this work could include news approaches to binary option pricing in practice, based on historical risk levels of relative entropy. Additionally, the joint dependence structure captured by relative entropy could shine new light on the co-movements and relative behaviours of binary and fixed-return options. As the size of the derivatives market grows, new and alternative risk measures, like relative entropy, will be increasingly sought after for managing the risk of option portfolios. Beyond the derivatives market, relative entropy can have many applications for any type of investment or structured product with discrete, fixed returns, and creates an opportunity to bring novel risk mitigation tools to a variety of industries.

Most of the literature on the topic of measuring such risk has focused primarily on the management of bet sizes, and this is usually driven solely by the expected probability of success or failure. The industry standard method for this type of capital allocation is the Kelly criterion (Kelly, 1956) [5]. This theory is reviewed in Section 2. Alternative methods for evaluating the risk of discrete portfolios are discussed in Section 3. The rest of this paper is arranged as follows. Section 4 demonstrates how relative entropy is the ideal convex risk measure for making quantitative portfolio allocation decisions for gambling wagers, and introduces a new family of entropic portfolio optimization problems, called discrete entropic portfolio optimization (DEPO). An example of DEPO applied to foreign exchange binary options is shown in Section 5 with the results compared to the leading Kelly criterion methods. To illustrate how DEPO is applied to a portfolio of gambling wagers, Section 5 further shows a sportsbook portfolio for the NFL 2019-20 season whle using DEPO for portfolio selection. Section 6 discusses the main conclusions derived from this work.

### Literature Review

Research on the topic of this type of portfolio optimization can be classified into two separate but related categories: gambling portfolios and investment portfolios. Some authors tailored their papers into one topic or the other, while other authors suggested that their strategies were equally applicable in both of them. Most of the research to date has solely focused on mathematics of the reward, i.e., maximizing the wealth, but contributed little work to the evaluation of risk. Kelly (1956) [5] discovered that a gambler’s exponential growth rate of their capital is maximized at the rate of transmission of information over that channel (the Shannon entropy using base two logarithms), and provided the Kelly criterion defining the optimal bet size to achieve such maximal growth. Further work on the Kelly criterion has been carried out and today it is widely used in investment theory as a standard bet size methodology by gamblers and investors alike, including even Warren Buffet (Benello, 2016) [8].

Applications to the investment securities became quite popular around in the 90s, with early work from Rotundo and Thorp (1992) [9] that applied the Kelly criterion to the U.S. stock market. Browne (1996) [10] derived an optimal gambling and investment policy for general stochastic processes using a continuous-time analog involving Brownian motion. After applying to blackjack and other gambling games, MacLean and Thorp (2010) [11] extended the Kelly criterion and its main variants such as fractional Kelly to applications in the securities market. Das (2016) [12] linked the Kelly criterion to portfolio optimization in the review of Browne (1996) [10], and Lavinio (2000) [13] applied it to a day-trading portfolio by using the *d*-ratio, or gain-loss ratio. O’Shaughnessy (2012) [14] suggested using correlated events to gain an edge over bookmakers by combining “for” and “against” bets in win-draw-loss markets. Taking uncertainty into account, Baker (2013) [15] shrank the Kelly bet sizes in order to compensate for the prediction uncertainty (a modified Kelly approach), which showed an improvement over the "raw" Kelly criterion. The Kelly criterion was analyzed further and demonstrated to be incredibly effective over time by MacLean (2013) [16]. Sinclair (2014) [17] devised a confidence interval for the Kelly criterion by calculating variance of the estimated Kelly criterion ratios. Applied to securities, Davari-Ardakani (2016) [18] developed a multistage optimization method that utilizes options to dynamically mitigate the market risk of an investment portfolio. Faias (2017) [19] optimized European option portfolios by proposing a myopic objective function to overcome limitations due to non-normality and small sample sizes encountered by traditional portfolio selection methods. Chu (2018) [20] recently introduced another fractional Kelly method that was based on the uncertainty of success probabilities by exploring various loss functions. Most recently, Hubacek (2019) [21] exploited sport-betting markets using a betting strategy that maximizes model prediction accuracy and minimizes model correlation with published bookmaker predictions. In most very recent research in the form of a working paper, Vecer (2020) [22] explores much similarly the use of Kullback–Leibler divergence as the optimal utility for the likelihood ratio of the densities corresponding to market takers and market makers. In this case it is for the purposes of determining optimal payoff functions and equilibrium for Arrow-Debreu securities (contracts that agree to pay one unit of a currency or commodity if a particular event occurs and zero otherwise, much like the concept of a binary option). Here Kullback–Leibler divergence is measured between the distribution of the market agent and the market equilibrium distribution, but not directly applied as a risk measure for purposes of portfolio optimization.

To date, there are not any suggestions for measuring or managing the risk of these option portfolios in literature. We will tackle it in this paper. The proposed DEPO mitigates the risk of a binary option portfolio by assessing the relative entropy of the portfolio returns. Additionally, DEPO maximizes the exponential growth rate of the portfolio by extending the Kelly criterion to multiple assets.

## 2. Maximum Exponential Growth Rate

### 2.1. The Kelly Criterion

In probability theory, the Kelly criterion (Kelly, 1956) [5] gives the bet size conditions required for gambling wagers to almost surely achieve the maximum exponential growth rate of wealth (or “bankroll”) based on assumed probability of success if the wager were to be placed repeatedly ad infinitum. For the purposes of this paper, we will just be concerned with the case that wagers that are paying rewards equal to the bet size, known as fair wagers, but intended further work on the topic that includes extending this to the generalized case of any size payout and odds. A short summary of the Kelly criterion is provided here, courtesy of Khanna (2016) [23]. Consider a wager with expected probability of success *p*, and expected probability of failure (1−p). After *N* trials, we denote the number of successes by *S* and number of failures by *F* (such that S+F=N). Let *w* represent the percentage of the portfolio balance to be wagered on each trial. Subsequently, for a starting portfolio balance $P_{0}$ and resulting portfolio balance $P_{N}$ after *N* trials, we have the following expression in (1),

(1) $$P_{N}=P_{0}{\left(1+w\right)}^{S}{\left(1-w\right)}^{F}.$$

It follows that $e^{N\log{\left(P_{N}/P_{0}\right)}^{\left(1/N\right)}}=\frac{P_{N}}{P_{0}}$, which implies that $\log{\left(\frac{P_{N}}{P_{0}}\right)}^{\left(1/N\right)}$ measures the exponential growth rate of wealth per trial. The Kelly criterion achieves the maximum expectation of this function via the growth rate coefficient *G*, defined as the expectation of the exponential growth rate per trial in (2),

(2) $$G\left(w\right)=E\left[\log{\left(\frac{P_{N}}{P_{0}}\right)}^{\left(1/N\right)}\right]=E\left[\frac{S}{N}\log\left(1+w\right)+\frac{F}{N}\log\left(1-w\right)\right]$$

As the experiment is repeated *N* times, *S* becomes a binomial random variable with parameters (N,p) and mean E(S)=np. Analogously, *F* also becomes a binomial random variable with parameters (N,1−p) and mean E(F)=n(1−p). Therefore by the additive property of expectations, G(w) can be expressed as

(3) G(w)=plog(1+w)+(1−p)log(1−w).

In order to maximize G(w) from (3), we take the first derivative with respect to *w* and set equal to zero, which yields the optimal bet size $w^{*}=2p-1$. It can be verified that $w^{*}$ is in fact a maximum by observing that the second derivative is negative at $w^{*}$, i.e., $\partial^{2}/\partial w^{2}G\left(w^{*}\right)<0$, and thus $w^{*}$ is a local maximum. Additionally, since $w^{*}$ is the only critical point and G(0)=0 while G(w)→−∞ as $x\to 1^{-}$, we can confirm that $w^{*}$ is a global maximum. Therefore, the bet size that maximizes the growth rate of wealth is $w^{*}=2p-1$, as a percentage of portfolio balance.

### 2.2. Extension of the Kelly Criterion to Multiple Wagers

Extensions to the Kelly criterion for multiple wagers have not really been extensively explored, beyond a brief expression for two independent wagers as shown by MacLean and Thorp (2010) [11]. The joint entropy of multiple wagers is only logically defined for equally-sized wagers, so for the scope of this paper we can restrict analysis to portfolios that contain equally-sized bet allocations, as can be seen in Section 4. With this constraint as a foundation, we can now generalize the Kelly criterion for two independent wagers and subsequently extend it to *n* wagers. Consider a collection of two independent events $Y_{1}$ and $Y_{2}$, with probabilities of success $p_{1}$ and $p_{2}$, respectively. Let *w* represent the total percentage of bankroll to be wagered. For a portfolio that allocates the wager equally across amongst events, the growth rate coefficient *G* would be

(4) $$\begin{array}{cc} G(w) & =\frac{1}{2}\left[p_{1}\log\left(1+w\right)+\left(1-p_{1}\right)\log\left(1-w\right)\right]+\frac{1}{2}\left[p_{2}\log\left(1+w\right)+\left(1-p_{2}\right)\log\left(1-w\right)\right]\\ \; & =\left(\frac{p_{1}+p_{2}}{2}\right)\log\left(1+w\right)+\left(1-\frac{p_{1}+p_{2}}{2}\right)\log\left(1-w\right). \end{array}$$

Therefore, by denoting $\bar{p}=\frac{p_{1}+p_{2}}{2}$ in (4) (this can be thought of as a blended probability of success), the Kelly criterion can be used here to identify the optimal size wager for maximum growth rate as $w^{*}=2\bar{p}-1=p_{1}+p_{2}-1$.

This expression can easily be extended to a portfolio of *n* wagers. For events $Y_{1},\ldots ,Y_{n}$ with success probabilities $p_{1},\ldots ,p_{n}$, it follows in (5) that

(5) $$G\left(w\right)=\left(\frac{1}{n}\sum_{i=1}^{n}p_{i}\right)\log\left(1+w\right)+\left(1-\frac{1}{n}\sum_{i=1}^{n}p_{i}\right)\log\left(1-w\right),$$

with growth rate maximized at $w^{*}=2\bar{p}-1$ for $\bar{p}=\frac{1}{n}\sum_{i=1}^{n}p_{i}$.

## 3. Shannon Entropy of Discrete Returns

### 3.1. Investments Versus Wagers

As a measure of dispersion, variance is a better tool for measuring risk in situations where the magnitude of observed returns affects the severity of an investor’s gain or loss. It is not so much a question of whether you win or lose, but rather how much you win or lose. The dispersive nature of continuous returns make variance an excellent measure of risk for this purpose. Major losses are the least desired result, so they are effectively penalized on a squared scale.

As a measure of uncertainty, both entropy and relative entropy are more suited for measuring risk for investment strategies where the magnitude of observed random variables do not affect the severity of an investor’s gain or loss, for example the discrete outcomes of gambling wagers. Consider a sports wager on the winner of a football game that pays 2 to 1 for a given bet in the sense for $1 wagered, a loss forfeits the $1 wager and a win returns $2 ($1 winnings plus the original $1). In the event of a loss, the outcome is −100%, i.e., the severity of the loss is invariant to whether the team loses by 1 point or 20 points. Equivalently for a win, the outcome is +100%, i.e., the severity of the gain is unchanged. In these situations, the information entropy of historical outcomes is more informative than variance, because entropy measures the level of randomness in these returns, and is not skewed by the underlying events that have no affect on the magnitude of the returns.

### 3.2. Joint Entropy of a Portfolio of Discrete Return Assets

It is first necessary to establish some theory required in order to calculate the risk of a portfolio of discrete return assets. Here we start with measuring the entropy and joint entropy of a discrete returns. A portfolio containing just one binary asset (for example, a wager) exhibits a special case of the joint entropy calculation, the univariate Shannon entropy (1948) [2,3] *H*. For a discrete random variable *X* with probability mass function P(·) of taking on values $x_{1},\ldots ,x_{n}$, the Shannon entropy *H* is the average amount of information produced by *X*, defined in (6) as

(6) $$H\left(X\right)=E\left(-\log P\left(X\right)\right)=-\sum_{k=1}^{m}P\left(x_{k}\right)\log P\left(x_{k}\right).$$

For a portfolio with more than one asset, the average amount of information is represented by the joint entropy. For *n* discrete random variables $X_{1},\ldots ,X_{n}$ with $m_{1},\ldots ,m_{n}$ probability respective states, the joint entropy $H(X_{1},\ldots ,X_{n})$ is given by (7),

(7) $$H\left(X_{1},\ldots ,X_{n}\right)=-\sum_{k_{1}=1}^{m_{1}}\cdots \sum_{k_{n}=1}^{m_{n}}P_{n}\left(x_{1k_{1}},\ldots ,x_{nk_{n}}\right)\log P_{n}\left(x_{1k_{1}},\ldots ,x_{nk_{n}}\right),$$

for joint probability distribution $P_{n}$. The joint entropy of event outcomes is the main discriminator of risk in the DEPO method for portfolio selection presented in this paper. Greater joint entropy (more fair randomness) represents lesser relative risk.

In the case of a discrete returns portfolio, each asset $R_{i}$ in the portfolio has a corresponding vector of historical outcomes $r_{ij}$, for i∈{1,…,n} and j∈{1,…,T}. For a binary option portfolio, these asset returns $r_{ij}$ can be either +1 or −1 (for +100% or −100% return outcomes corresponding to success and failure, respectively). Subsequently, the portfolio outcome at each point in time can be represented by the cross-sectional vector $r_{\cdot j}=\left(r_{1j},\ldots ,r_{nj}\right)$. These are the event outcomes to be used in the portfolio calculation for joint entropy.

## 4. Minimum Relative Entropy

For the purposes of DEPO, the risk of an option portfolio is defined here as the relative entropy of portfolio returns, with respect to the uniform distribution. We start by providing some background theory on the topic here, and attest to its value as a portfolio risk measure.

### 4.1. Kullback–Leibler Divergence

The Kullback–Leibler divergence [6,7] measures the distance (or more specifically, the directed divergence) between two probability distributions. If *P* and *Q* are two discrete distributions with the support X, the Kullback–Leibler divergence between them, also known as the relative entropy of *P* with respect to *Q*, is given by (8),

(8) $$\begin{array}{cc} D_{KL}\left(P\;∥\;Q\right) & =-\sum_{x\in X}P\left(x\right)\log\left(\frac{Q(x)}{P(x)}\right)\\ & =\;\;\;\;\sum_{x\in X}P\left(x\right)\log\left(\frac{P(x)}{Q(x)}\right). \end{array}$$

Gibbs’ inequality (see Mackay, 2003) [24] shows that the relative entropy is always non-negative, as shown in (9),

(9) $$D_{KL}\left(P\;∥\;Q\right)\geq 0,$$

with equality if and only if P=Q almost everywhere.

The use of relative entropy requires the existence of a target distribution—a distribution from which the observed distribution is measured. Minimizing the relative entropy ensures the observed distribution resembles the target distribution as closely as possible. In the case of a fair betting wager, we argue that the desired target distribution is the uniform distribution.

Let *X* be a discrete random variable with probability mass function *P*, whose Shannon entropy relates to the relative entropy. It is shown in (10) that the Shannon entropy of *X* is equal to the entropy of the *m*-state discrete uniform distribution $U_{m}$ (maximum entropy) less the relative entropy a discrete distribution *P* with respect to $U_{m}$,

(10) $$H\left(X\right)=\log\left(m\right)-D_{KL}\left(P\;∥\;U_{m}\right)\Rightarrow D_{KL}\left(P\;∥\;U_{m}\right)=\log\left(m\right)-H\left(X\right).$$

Using the same combinatorial technique that was employed for REPO [1], the Shannon entropy of portfolio returns can be estimated empirically via probability generating functions. For a collection of *n* discrete return assets over time period j=1,…,T, let $r_{j}=\left(r_{1j},\ldots ,r_{nj}\right)$ denote the cross-sectional *n*-dimensional vector of outcomes across one observational row of data, and let them be uniquely represented by the collection of $u_{k}$’s such that $u_{k}=\left\{r_{j}\;|\;r_{j}\neq u_{l},\mathrm{for}\;\mathrm{some}\;j,\;\mathrm{and}\;\mathrm{any}\;l\neq k\right\}$. Subsequently, the empirical Shannon entropy of option portfolio returns $R_{Q}$ can be expressed as in (11),

(11) $$H\left(R_{Q}\right)\approx -\sum_{k=1}^{m}\left(\frac{g^{\left(k\right)}\left(0\right)}{k!}\right)\log\left(\frac{g^{\left(k\right)}\left(0\right)}{k!}\right),$$

for *k*th-derivative at x=0 of generating function

(12) $$g\left(x;w_{1},\ldots ,w_{n}\right)=\frac{1}{T}\sum_{j=1}^{T}x^{\left\{k\;:\;r_{j}=u_{k}\right\}}.$$

Therefore, the risk of a binary option portfolio can be measured by the relative entropy of portfolio returns $R_{Q}$, estimated empirically as in (13),

(13) $$D_{KL}\left(R_{Q}\;∥\;U_{m}\right)\approx \log\left(m\right)+\sum_{k=1}^{m}\left(\frac{g^{\left(k\right)}\left(0\right)}{k!}\right)\log\left(\frac{g^{\left(k\right)}\left(0\right)}{k!}\right).$$

### 4.2. Relative Entropy as a Convex Risk Measure

As outlined by the Education and Examination Committee of the Society of Actuaries (Hardy, 2006) [25], a risk measure is a function ρ:X→R, for a linear space X and random variable X∈X, that maps a given loss distribution to the real number line. Risk measures are very popular in financial mathematics and actuarial science to quantify risk exposure, and are based on the so-called premium principles, the purpose of which is to establish an appropriate premium to charge for a given risk. Several common early premium principles include the equivalence principle, expected value principle, variance principle, and standard deviation principle. For example, the variance principle is given by ρ(X)=E(X)+kVar(X), for a fixed constant k≥0.

Connections between utility maximization and entropy in the context of risk measures have been previously explored in the literature, and a comprehensive review is presented in the text by Follmer and Schied (2011) [26]. Of particular note is the entropic risk measure $\rho_{\beta }$ given by $\rho_{\beta }\left(X\right)=\sup_{P}\left(E_{P}\left[-X\right]-\frac{1}{\beta }D_{KL}\left(P\;∥\;Q\right)\right)$, highlighting the connection between utility-based shortfall risk and divergence risk measures. To that end, here we would like to introduce the relative entropy principle, similar to that developed by Ahmadi-Javid (2016) [27], to evaluate the risk of portfolios based on (uniform) relative entropy. For X∈X according to a discrete distribution *P*, the relative entropy principle is given by (14),

(14) $$\rho \left(X\right)=E\left(X\right)+kD_{KL}\left(P\;∥\;U\right),$$

for the relative entropy $D_{KL}\left(P\;∥\;U\right)$ of *P* with respect to a discrete uniform distribution *U* that has the same support as *P*.

Further to the concept of risk measures, a convex risk measure (Artzner, 1999) [28] is a risk measure ρ:X→R that satisfies the following criteria for each X,Y∈X:

(i) Monotonicity. If X≤Y, then ρ(X)≤ρ(Y),
(ii) Translation invariance. If c∈R, then ρ(X+c)=ρ(X)+c, and
(iii) Convexity. ρ(λX+(1−λ)Y)≤λρ(X)+(1−λ)ρ(Y), for 0≤λ≤1.

We present some mathematical properties of relative entropy first, and then show that the relative entropy qualifies as a convex risk measure.

For independent distributions, relative entropy is additive similar to the way Shannon entropy is additive (Kullback, 1996) [29]. Let $P_{1},P_{2}$ be independent distributions with joint probability mass function $P\left(x,y\right)=P_{1}\left(x\right)P_{2}\left(y\right)$, and similarly let $Q_{1},Q_{2}$ be independent with $Q\left(x,y\right)=Q_{1}\left(x\right)Q_{2}\left(y\right)$, where $P_{i}$ and $Q_{i}$ have the same support for i=1,2. Subsequently, it follows that expression (15) holds true,

(15) $$D_{KL}\left(P\;∥\;Q\right)=D_{KL}\left(P_{1}\;∥\;Q_{1}\right)+D_{KL}\left(P_{2}\;∥\;Q_{2}\right).$$

**Proposition** **1.** *Based on the entropy principle, relative entropy satisfies the three conditions required to be considered a valid convex risk measure: (i) monotonicity, (ii) translation invariance, and (iii) convexity.*

**Proof.**  Let ρ(·) be a risk measure based on the stated relative entropy principle, such that for constant k≥0 and X∈X following discrete distribution *P*, ρ is of the form $\rho \left(X\right)=E\left(X\right)+kD_{KL}\left(P\;∥\;U\right)$.

(i) Monotonicity. For a risk measure ρ(·) to be monotonic it must satisfy: If X,Y∈X and X≤Y almost surely then ρ(X)≤ρ(Y) almost surely. Using ρ(X) as stated, we have $\rho \left(X\right)=E\left(X\right)+kD_{KL}\left(P\;∥\;U\right)$ and $\rho \left(Y\right)=E\left(Y\right)+kD_{KL}\left(Q\;∥\;U\right)$ for discrete distributions *P* and *Q*. Because X≤Y implies $D_{KL}\left(P\;∥\;U\right)\leq D_{KL}\left(Q\;∥\;U\right)$ as a consequence of the data processing inequality (Cover, 1991) [30], it follows that
  (16) $$\rho \left(X\right)=E\left(X\right)+kD_{KL}\left(P\;∥\;U\right)\leq E\left(Y\right)+kD_{KL}\left(Q\;∥\;U\right)=\rho \left(Y\right),$$
  almost surely. Therefore, (16) shows ρ is monotonic.
(ii) Translation invariance. For a risk measure ρ(·) to exhibit translation invariance it must satisfy: If X∈X then ρ(X+c)=ρ(X)+c. Recall the risk measure based on the relative entropy principle is of the form $\rho \left(X\right)=E\left(X\right)+kD_{KL}\left(P\;∥\;U\right)$. Since H(X+c)=H(X), for all *c*, it follows that $D_{KL}\left(P\left(X\right)\;∥\;U\right)=D_{KL}\left(P\left(X+c\right)\;∥\;U\right)$ and, thus, we have (17),
  (17) $$\rho \left(X+c\right)=E\left(X+c\right)+kD_{KL}\left(P\left(X+c\right)\;∥\;U\right)=E\left(X\right)+c+kD_{KL}\left(P\left(X\right)\;∥\;U\right)=\rho \left(X\right)+c.$$
  Therefore ρ exhibits translation invariance.
(iii) Convexity. A risk measure ρ(·) is convex if: For $Z_{1},Z_{2}\in X$ and λ∈[0,1] it follows that: $\rho \left(\lambda Z_{1}+\left(1-\lambda \right)Z_{2}\right)\leq \lambda \rho \left(Z_{1}\right)+\left(1-\lambda \right)\rho \left(Z_{2}\right)$. It is known that $D_{KL}\left(P\;∥\;Q\right)$ is convex in the pair of probability mass functions (P,Q). If $\left(P_{1},Q_{1}\right)$ and $\left(P_{2},Q_{2}\right)$ are two pairs of probability mass functions, then (18) follows,
  (18) $$D_{KL}\left(\lambda P_{1}+\left(1-\lambda \right)P_{2}\;∥\;\lambda Q_{1}+\left(1-\lambda \right)Q_{2}\right)\leq \lambda D_{KL}\left(P_{1}\;∥\;Q_{1}\right)+\left(1-\lambda \right)D_{KL}\left(P_{2}\;∥\;Q_{2}\right).$$

Accordingly, (16)–(18) prove that ρ is a convex measure. And therefore, based on the relative entropy principle, relative entropy is a valid convex risk measure since all three necessary conditions are satisfied. □

### 4.3. Minimum Risk Option Portfolios with Relative Entropy

When selecting a portfolio of securities, the objective was to minimize the discrete entropy of portfolio returns, as seen in REPO [1] (i.e., to minimize entropy, and maximize expected returns). Low entropy means low risk. In contrast, when dealing with gambling outcomes the opposite is true, as maximum entropy proves to be the lowest risk option, due to the uniformity of gambling outcomes (as opposed to normality). This can be demonstrated in the following example. Consider an online casino website that offers a virtual roulette game. This roulette game is a black-box (its programming code or internal structure is unknown), but imagine that the site displays the entropy of the red or black outcomes over the past 100 spins. Take two independent roulette wheels, *A* and *B*. Wheel *A*’s past 100 spins were evenly split between red and black, uniformly 50/50, giving an entropy of 1-bit, the maximum entropy possible (using base 2 entropy, since we only have binary outcome here). Wheel *B*’s past 100 spins were 75 of one color and 25 of the other, for a (lesser) entropy of 0.8113 bits. Which wheel is lower risk?

Without knowing the color advantage of wheel *B*, clearly the fairness of wheel *A* is lower risk between the two. Arguably, even if one did know the 75/25 split was in favor of red, wheel *B* is still riskier. One may play on the “gambler’s fallacy” (Keren, 1994) [31] and bet on black with the conviction that some black spins must be forthcoming to even out the odds. Or one may assume that the wheel’s randomness is flawed and bet on red to capitalize on the fault. Both of the strategies carry much greater risk than placing any color bet on wheel *A*. Therefore it is evident that for uniform-type distributions, such as gambling wager outcomes, maximal entropy is the desired minimum-risk method.

As discussed in Section 4.2, based on the relative entropy princple, the relative entropy is proven to be a convex risk measure. Thinking back to the REPO [1] problem, the goal of the objective function was to minimize the portfolio entropy. Here we wish to minimize the portfolio relative entropy with respect to the uniform density, which means that the goal is to obtain as close to a uniform distribution as possible. This would be analogous to maximizing the portfolio entropy in REPO, but this proves to be the minimal risk portfolio for a collection of gambling wagers in DEPO.

One interesting point to note is that DEPO assigns an equally-weighted allocation across chosen assets. This is due to the nature of joint entropy and joint relative entropy. Joint entropy is a measurement determined strictly by the inclusion or exclusion of random variables. Consequently, a fractional inclusion of a random variable contributes the same amount of marginal entropy as the full inclusion, by the property of joint entropy $H\left(aX_{1},bX_{2}\right)=H\left(X_{1},X_{2}\right)$, for any 0<a,b≤1. Therefore, while the total allocation is determined by the growth rate objective function, it is split equally amongst the selected assets in the portfolio. As seen in recent works, such as Low et al. (2016) [32], the equally-weighted strategy still remains difficult to outperform in the portfolio selection problem.

### 4.4. Discrete Entropic Portfolio Optimization (DEPO)

The new discrete entropic portfolio optimization (DEPO) problem uses a multi-objective function that minimizes the empirical relative entropy and maximizes expected growth rate. Using this optimization, users can make portfolio selections based on a chosen risk tolerance. The highest risk portfolio solely maximizes the expected portfolio growth rate, which is equivalent to the Kelly criterion method. The lowest risk portfolio minimizes the portfolio relative entropy, which is the most diversified portfolio allocating capital to all *n* options equally. Somewhere in between lies a user’s optimal portfolio of choice. For the two-state case of binary options we would have a series of events with possible outcome states *L* (loss) and *W* (win), which can be denoted as outcomes u∈{0,1}. This leads to the simplified DEPO problem. Consider *n* events with potential investment opportunities. Let $p_{i}$ represent the probabilities of success for event i∈{1,…,n}, and let $w_{i}$ represent the percentage of portfolio to be allocated to event *i*, with the total portfolio allocation summing to $\omega =w_{1}+\cdots +w_{n}$. Let $r_{j}=\left(r_{1j},\ldots ,r_{nj}\right)$ denote the cross-sectional *n*-dimensional vector of outcomes across one observational row of data. Over *T* data points this leads to *m* historical unique vectors of 0’s and 1’s, $u_{k}=\left\{r_{j}\;|\;r_{j}\neq u_{l},\mathrm{for}\;\mathrm{some}\;j,\;\mathrm{and}\;\mathrm{any}\;l\neq k\right\}$ for k=1,…,m such that each $u_{k}$ is unique, with *m* bounded by either *T* or the maximum number of possible combinations $\lambda^{n}$, so $m=min(T,\lambda^{n})$. Basically, the collection of $u_{k}$’s is a unique representation of the $r_{j}$’s with no duplicates. Let us also denote $\eta =\sum_{i=1}^{n}I\left(w_{i}\right)\leq n$ as the number of chosen options in the portfolio, where $I(w_{i})$ is the indicator function for the event $\left\{w_{i}>0\right\}$. Then the DEPO problem is defined as the following optimization program in (19) (using logarithm base 2, since we have binary event outcomes *u*),

(19) $$\begin{array}{cc} \mathrm{minimize}\;\; & D_{KL}\left(R_{Q}\;∥\;U_{m}\right)=\log_{2}\left(m\right)+\sum_{k=1}^{m}\left(\frac{g^{\left(k\right)}\left(0\right)}{k!}\right)\log_{2}\left(\frac{g^{\left(k\right)}\left(0\right)}{k!}\right)\\ \mathrm{maximize}\;\; & G\left(\omega \right)=\left(\frac{1}{\eta }\sum_{i=1}^{n}I\left(w_{i}\right)p_{i}\right)\log_{2}\left(1+\omega \right)+\left(1-\frac{1}{\eta }\sum_{i=1}^{n}I\left(w_{i}\right)p_{i}\right)\log_{2}\left(1-\omega \right),\\ \mathrm{subject}\;\mathrm{to}\;\; & \omega =w_{1}+\cdots +w_{n}\leq 1,\;\;\;\\ & w_{i}\geq 0\;\;\forall \;i,\\ & w_{i}=w_{j}=\eta^{-1}\omega \;\;\forall \;\left\{\left(i,j\right):I\left(w_{i}\right)=I\left(w_{j}\right)=1\right\}, \end{array}$$

for the *m*-state uniform distribution $U_{m}$ and *k*th-derivative at x=0 of probability generating function

(20) $$g\left(x;w_{1},\ldots ,w_{n}\right)=\frac{1}{T}\sum_{j=1}^{T}x^{\left\{k\;:\;\left(I\left(w_{1}\right)r_{1j},\ldots ,I\left(w_{n}\right)r_{nj}\right)=u_{k}\right\}}.$$

The last constraint in the optimization problem (19) stems from the fact that joint entropy measures randomness strictly based on the inclusion or exclusion of a random variable. The joint entropy calculation is completely indifferent to the size of allocation, so any non-zero weight $w_{i}$ contributes the corresponding marginal entropy from asset *i*, regardless of the magnitude of $w_{i}$. For this reason every asset included in the portfolio is assigned an equal weighting of $\eta^{-1}\omega$.

### 4.5. Risk-Adjusted Performance

Now that a risk–reward framework for finding optimized portfolios has been established, we can calculate a risk-adjusted return ratio for comparing portfolios growth rates of different risk profiles. This is analogous to the “reward-to-variability ratio” by Sharpe (1966) [33], better known as the Sharpe ratio. The Sharpe ratio of a portfolio is defined in (21),

(21) $$S_{n}=\frac{E(R_{a}-R_{b})}{\sigma_{a}}=\frac{E(R_{a}-R_{b})}{\sqrt{\mathrm{Var}(R_{a}-R_{b})}},$$

where $R_{a}$ is the portfolio return, $R_{b}$ is the risk-free rate of return, and $\sigma_{a}$ is the standard deviation of the portfolio excess return.

As shown by Eling (2008) [34] and Rad et al. (2016) [35], for returns that are not normally distributed it is well known that the Sharpe ratio has the potential to underestimate risk, thereby overestimating the risk-adjusted performance. The authors employed methods, such as lower partial moment measures and drawdown measures, to circumvent this bias. To that end, here we introduce the risk-adjusted ratio for comparing growth rates of discrete return portfolios, called the Growth Rate Over UNiform Divergence (GROUND) ratio. This ratio measures the expected growth rate of a portfolio, adjusted by its risk level—relative entropy with respect to the uniform distribution. Let $U_{m}$ be the *m*-state discrete uniform distribution. For chosen portfolio $R_{a}$ and minimum risk portfolio $R_{b}$ existing in the *m*-state event space, the GROUND ratio $\Gamma_{m}$ is defined by (22),

(22) $$\begin{array}{cc} \Gamma_{m} & =\frac{E(G_{a}\left(\omega_{a}\right)-G_{b}\left(\omega_{b}\right))}{D_{KL}\left(R_{a}\;∥\;U_{m}\right)-D_{KL}\left(R_{b}\;∥\;U_{m}\right)}\\ & =\frac{E(G_{a}\left(\omega_{a}\right)-G_{b}\left(\omega_{b}\right))}{H\left(R_{b}\right)-H\left(R_{a}\right)}, \end{array}$$

where $G_{a}\left(\omega_{a}\right)$ is the growth rate of the chosen portfolio with weighting $\omega_{a}$, $G_{b}\left(\omega_{b}\right)$ is the growth rate of the minimum risk portfolio with weighting $\omega_{b}$, $D_{KL}\left(R_{a}\;∥\;U_{m}\right)$ is the relative entropy of the chosen portfolio with respect to $U_{m}$, $D_{KL}\left(R_{b}\;∥\;U_{m}\right)$ is the relative entropy of the minimum risk portfolio with respect to $U_{m}$, and H(·) is the Shannon entropy.

## 5. Portfolio Selection Examples with DEPO

### 5.1. FOREX Binary Option Portfolio Example

#### 5.1.1. Data

In this example, actual binary option data are presented from the ten foreign exchange currency pairs available for trading on the North American Derivatives Exchange (NADEX), which can be found at www.NADEX.com. All of the historical intraday contract prices, strikes, and outcomes are from the time period January 2019 to January 2020, totalling 346,760 historical trades. Daily option contracts can expire at intervals of four hours, namely 3:00 a.m., 7:00 a.m., 11:00 a.m., 3:00 p.m., 7:00 p.m., and 11:00 p.m. Note that one can bet either “for” or “against” each option (buy or sell). Each currency pair has a series of available option contracts to choose from, with estimated success rates ranging anywhere from 5% to 95%. Note that all probabilities for success rates of these options are only estimates based on market consensus. For the purposes of this paper, we focus solely on options with an estimated success rate of around 50% in order to ensure the expected win payoff is close to the amount wagered, so we restrict the data to observations with market consensus of 45% to 55% estimated probability of expiring in-the-money. This results in 10,435 remaining observations encompassing 1332 possible contract expiry dates for each currency pair. Intraday option contract outcomes versus historical strike prices are recorded and computed as follows in (23),

(23) $$I\left(E_{P}>S_{P}\right)-I\left(E_{P}\leq S_{P}\right),$$

where $E_{P}$ is the currency pair expiration price and $S_{P}$ is the strike price of the option (the minimum price necessary to become in-the-money). The result is 1 or −1 for respectively in-the-money or out-the-money, and 0 for an unavailable trade. Using this historical data, we are able to empirically calculate the estimated relative entropy for each option. The binary options and their respective outcomes against strike prices over January 2019 to January 2020 are presented below in Table 1, together with their estimated relative entropies.

For the month of February 2020 there are 110 short-term FOREX binary option contract expiration periods, and projections (estimates) for each option are presented from NADEX market consensus probability of expiring above the strike price (in-the-money). These projections measure with what probability market bettors are expecting currency pairs to land in-the-money, as explained in the NADEX lessons page [36]. At each contract expiration period, there are ten available currency pairs to bet on, as shown in Table 1, and the historical results summarized there are used to evaluate the estimated relative entropy risk of each option. The emulation here shows how DEPO performs against leading Kelly criterion methods for picking a portfolio of options at each contract expiration over the course of February 2020.

For illustrative purposes, let us examine this method applied to the first contract expiry period, Sunday 2 February 2020 11:00 p.m. (as FOREX markets are open globally 24 h a day during the weekdays, this period is the first available contract expiry in February, since markets have already opened Monday morning in Asia). Table 2 lists the details of select currency pair binary options, with contract strike price and the market consensus estimates for each option.

DEPO determines which collection of options to buy or sell and what percentage of portfolio to allocate to each, in order to build the optimal risk–reward binary option portfolio.

Each potential portfolio has an expected growth rate given the consensus estimates, and an estimated relative entropy with respect to the uniform distribution, empirically calculated over the historical data. The historical data contains T=1332 data points for each currency pair, and the total number of combinations for n=10 binary outcomes is $2^{10}=1024$, so the maximum joint entropy that can possibly be exhibited is $\log_{2}\left(2^{n}\right)=10$, corresponding to the uniform distribution $U_{m}$ with m=1024 possible probability states. Therefore, as shown in (24), a portfolio $R_{Q}$ with joint entropy $H(R_{Q})$ has an estimated relative entropy of

(24) $$\begin{array}{cc} D_{KL}\left(R_{Q}\;∥\;U_{m}\right) & =\log_{2}\left(2^{n}\right)-H\left(R_{Q}\right)\\ \; & =10-H(R_{Q}). \end{array}$$

#### 5.1.2. Efficient Frontier and Portfolio Selection

**Figure 1:**
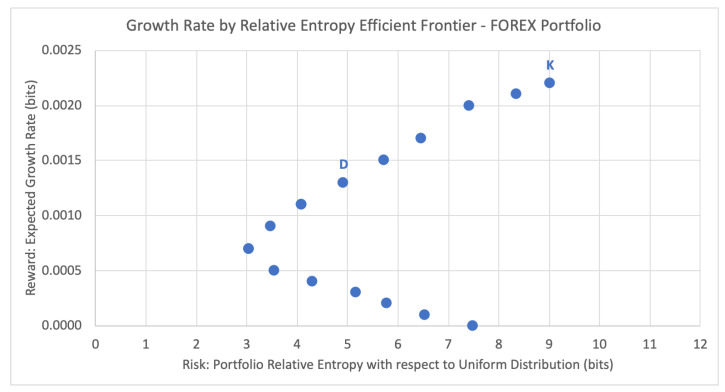
Growth rate by relative entropy efficient frontier for FOREX binary option portfolio.

In the portfolio selection problem, the efficient frontier refers to the set of optimal portfolios that yield the greatest expected return for a defined level of risk, or equivalently the least risk for a defined level of expected return (the dual problem). The efficient frontier illustrates the risk–return trade-off for a given set of optimal portfolios. Here we show the analogous efficient frontier for portfolios with discrete return assets, when comparing the expected growth rates and risk levels (estimated relative entropy) of each efficient portfolio. For the same contract expiry period of 2 February 2020 11:00 p.m., Figure 1 below plots the potential portfolios and their respective expected growth rates against their inherent risk profile, the estimated relative entropy with respect to the uniform distribution Uniform($2^{n}$) of historical joint outcomes of the portfolio.

For the current month emulation, the Kelly criterion strategy chooses the portfolio $R_{K}$ that maximizes the expected growth rate. For the first contract expiration period, this leads to the top right-most data point K = (9.0198, 0.0022), with estimated relative entropy of 9.0198 and expected growth rate of 0.0022. This portfolio consists of just the last currency pair listed in Table 2, selling USD/JPY > 108.40 to land out-the-money with 52.75% probability of success. According to the Kelly criterion, the optimal bet size here is 2p−1=2×(0.5275)−1=0.055 and, thus, the chosen portfolio for this period is using 5.5% of portfolio funds to sell USD/JPY > 108.40, as shown in Table 3. This strategy disregards any concept of risk associated with the expected portfolio growth rate of G(ω)=0.0022.

Alternatively, DEPO chooses the optimal portfolio based on the risk–reward trade-off. For each of the 1332 contract expiration periods, portfolio selection is performed according to the following DEPO problem (25) with n=10 and T=1332,

(25) $$\begin{array}{cc} \mathrm{maximize}\;\; & G\left(\omega \right)=\left(\frac{1}{\eta }\sum_{i=1}^{n}I\left(w_{i}\right)p_{i}\right)\log_{2}\left(1+\omega \right)+\left(1-\frac{1}{\eta }\sum_{i=1}^{n}I\left(w_{i}\right)p_{i}\right)\log_{2}\left(1-\omega \right),\\ \mathrm{subject}\;\mathrm{to}\;\; & D_{KL}\left(R_{Q}\;∥\;U_{m}\right)=\log_{2}\left(2^{n}\right)+\sum_{k=1}^{T}\left(\frac{g^{\left(k\right)}\left(0\right)}{k!}\right)\log_{2}\left(\frac{g^{\left(k\right)}\left(0\right)}{k!}\right)\leq 5,\\ & \omega =w_{1}+\cdots +w_{n}\leq 1,\\ & w_{i}\geq 0\;\;\forall \;i,\\ & w_{i}=w_{j}=\eta^{-1}\omega \;\;\forall \;\left\{\left(i,j\right):I\left(w_{i}\right)=I\left(w_{j}\right)=1\right\}, \end{array}$$

for Uniform($2^{n}$) as the target distribution, and for the *k*th-derivative at x=0 of probability generating function

(26) $$g\left(x;w_{1},\ldots ,w_{n}\right)=\frac{1}{T}\sum_{j=1}^{T}x^{\left\{k\;:\;\left(I\left(w_{1}\right)r_{1j},\ldots ,I\left(w_{n}\right)r_{nj}\right)=u_{k}\right\}}.$$

While the Kelly criterion places the entire allocation on the option (or options) with the greatest estimated probability of success, DEPO diversifies the portfolio by distributing the percent allocation across multiple options according to the appropriate risk profile. For period 1, DEPO selects data point D = (4.9264, 0.0013), with estimated relative entropy of 4.9264 and expected growth rate of 0.0013. This corresponds to the optimal portfolio of six option contracts listed below in Table 4, with a total portfolio allocation of 4.2% (as compared to the 5.5% allocated by the Kelly criterion).

Portfolio efficiency can be measured using the risk-adjusted GROUND ratio. In regards to the risk-adjusted expected returns, the DEPO portfolio has a GROUND ratio of $\Gamma_{m}=\left(0.0013-0.0007\right)/\left(4.9264-3.0546\right)=0.0321\%$, over 25% more efficient than the Kelly criterion portfolio at $\Gamma_{m}=\left(0.0022-0.0007\right)/\left(9.0198-3.0546\right)=0.0251\%$.

The actual results that follow this period saw USD/JPY expire at 108.23, below the strike price of 108.40. Therefore the Kelly criterion strategy experiences a gain of 5.5% of portfolio balance in period 1. In regards to the DEPO portfolio, gains from USD/JPY and AUD/JPY sells expiring out-the-money were offset by USD/CAD and USD/CHF expiring in-the-money (losing both sell options) and EUR/USD and GBP/USD expiring out-the-money (losing both buy options), for a total loss of 1.4% in period 1.

#### 5.1.3. Comparison to the Kelly Criterion over Time

**Figure 2:**
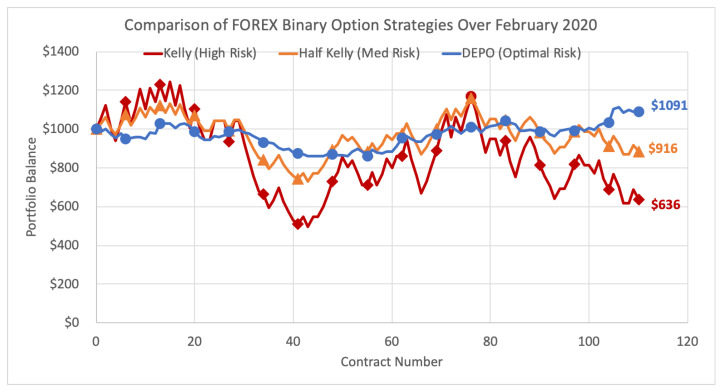
Comparison of FOREX binary option strategies over February 2020.

Here we demonstrate the performance of DEPO versus the Kelly and Kelly-variant strategies over the full month February 2020 of FOREX binary options on NADEX. Methods in the previous Section 5.1.2 are repeated multiple times per day at four-hour interval contract expiry times. The Kelly criterion strategy wagers the optimal bet allocation each period on the option (or options) that have the greatest estimated probability of success. The half Kelly is the same strategy, but utilizes the fractional Kelly-variant by wagering half the Kelly criterion bet size on the same options. DEPO optimal risk strategy uses the DEPO algorithm each period to select the portfolio with the greatest expected growth rate subject to the main constraint that the portfolio has estimated relative entropy of no greater than 5. Each strategy began the month with $1000 and the total results are shown below in Figure 2.

While the Kelly and half Kelly strategies show massive variability with large portfolio highs and lows, DEPO remains consistently stable and it ends the month with a modest profit of 9.1%, up $91. The half Kelly finishes the month at a loss of 8.4% to $916, and the full Kelly finishes at a significant loss of 36.4% to $636. As the top market consensus predictions perform negatively through contracts 30–40 as well as 80+, DEPO’s diversification generates consistent returns and builds on profits. The main purpose of DEPO is to mitigate risk of inaccurate predictions, and the goal is well accomplished.

**Figure 3:**
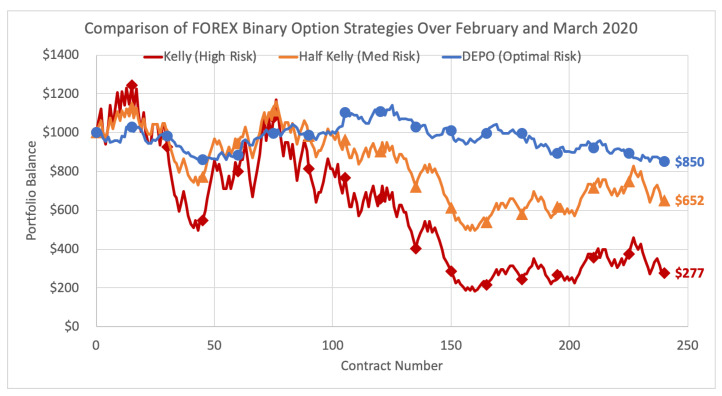
Comparison of FOREX binary option strategies over February and March 2020.

The extended emulation is even more telling. We continue emulating DEPO and the Kelly strategies over the month of March 2020, and while the Kelly criterion strategy begins to deteriorate rather drastically, DEPO strategy holds quite strong throughout the entire period and finishes the month with just a modest loss of 15% at $850. The half Kelly finishes at a loss of 34.8% to $652, and the full Kelly down a whopping 72.3% to only $277. The results are illustrated below in Figure 3.

### 5.2. NFL Sportsbook Example

#### 5.2.1. Data

In the example provided here, actual game data are presented for all 32 teams from the National Football League (NFL) over the past several seasons. Unfortunately, the NFL season is a prime example of extremely small sample size data. The regular season consists of only 17 weeks and teams play only 16 regular season games. This would normally pose a problem for traditional portfolio selection methods that are based on normality, but the non-parametric approach by DEPO makes it well capable of handling such small sample data. Using archived data from www.teamrankings.com/nfl and www.archive.org, we were able to gather eight full past seasons of NFL games with historical Las Vegas point spreads from 2011–12 to 2018–19 seasons, totaling 136 weeks of results. Weekly game outcomes versus historical points spreads were recorded and computed as follows in (27),

(27) $$I\left(M+S_{LV}>0\right)-I\left(M+S_{LV}<0\right),$$

where *M* is the team’s winning margin (negative in case of a loss), and $S_{LV}$ is the Vegas pre-game point spread (negative in case of a favourite). This results in 1 or −1 for respectively covering the spread or not, and 0 for a tie or week off. Using this historical data, we can empirically calculate the estimated relative entropy for each team. The teams and their respective outcomes versus spreads over the 2011–12 to 2018–19 seasons are presented below in Table 5.

Over the 17 weeks of the 2019–20 regular season, projections for each game were gathered from pre-game market betting consensus as presented on the www.covers.com/sports/nfl/matchups website. These estimates measure what percentage of market bettors are betting on either side of the Las Vegas spread line. Interestingly, over the course of the 17 week regular season, these market consensus estimates performed about as well as a coin-toss, with a weighted average of −0.73% versus actual outcomes (approximately 50% accuracy). Despite this, the market consensus estimates are unrealistically high. Therefore, in order to use more sensible estimates, as well as demonstrate the flexibility DEPO has to user inputs, we use posterior probabilities that are assigned as half of each team’s market consensus edge. The historical results summarized in Table 5 are used to evaluate the estimated relative entropy risk of each game. On each Sunday, there are between 11 and 16 games taking place. The emulation here shows how DEPO performs against the leading Kelly criterion methods for picking a portfolio of wagers on each of the 17 Sundays throughout the regular season.

For illustrative purposes, let us examine this method applied to the first week: Sunday 8 September 2019, which aired 13 NFL games in total. Table 6 lists the scheduled games, pre-game Vegas point spreads, and the market consensus estimates for each game.

Each game represents a potential bet opportunity, particularly betting on the team with higher consensus to cover the spread and betting on the team with lower consensus to not cover the spread. DEPO determines which collection of bets to select and what percentage of bankroll to wager on each, in order to build the optimal risk–reward sportsbook portfolio.

Each potential portfolio has an expected growth rate given the consensus projections, and an estimated relative entropy with respect to the uniform distribution, empirically calculated using the historical data. The historical data contain T=136 data points for each team, so the maximum joint entropy that can possibly be exhibited is $\log_{2}\left(T\right)=7.0875$, corresponding to a uniform distribution $U_{m}$ with m=136 possible probability states. Therefore, as shown in (28), a portfolio $R_{Q}$ with joint entropy $H(R_{Q})$ has an estimated relative entropy of

(28) $$\begin{array}{cc} D_{KL}\left(R_{Q}\;∥\;U_{T}\right) & =\log_{2}\left(T\right)-H\left(R_{Q}\right)\\ \; & =7.0875-H(R_{Q}). \end{array}$$

#### 5.2.2. Efficient Frontier and Portfolio Selection

For the same first week of Sunday 8 September 2019, Figure 4 below plots the potential sportsbook portfolios and their respective expected growth rates against their inherent risk profile, the estimated relative entropy with respect to the uniform distribution Uniform(*T*) of historical joint outcomes of the portfolio.

For the current season emulation, the Kelly criterion strategy chooses the portfolio $R_{K}$ that maximizes the expected growth rate. For week 1, this leads to the top right-most data point K = (5.089, 0.029), with estimated relative entropy of 5.089 and expected growth rate of 0.029. This portfolio consists of just the first game listed in Table 6, KC −3.5 @ JAC, taking KC to cover with a 60% probability of success (half the edge of the 70% market consensus). According to the Kelly criterion, the optimal bet size here is 2p−1=2×(0.6)−1=0.2, and thus the chosen portfolio for week 1 is 20% of bankroll on KC −3.5, as shown in Table 7. This strategy disregards any concept of risk associated with the expected portfolio growth rate of G(ω)=0.029.

Alternatively, DEPO chooses the optimal portfolio based on the risk–reward trade-off. For each of the weeks 1 to 17, portfolios selection is performed according the following DEPO problem (29), with *n* being the number of available games that Sunday and T=136,

(29) $$\begin{array}{cc} \mathrm{maximize}\;\; & G\left(\omega \right)=\left(\frac{1}{\eta }\sum_{i=1}^{n}I\left(w_{i}\right)p_{i}\right)\log_{2}\left(1+\omega \right)+\left(1-\frac{1}{\eta }\sum_{i=1}^{n}I\left(w_{i}\right)p_{i}\right)\log_{2}\left(1-\omega \right),\\ \mathrm{subject}\;\mathrm{to}\;\; & D_{KL}\left(R_{Q}\;∥\;U_{T}\right)=\log_{2}\left(T\right)+\sum_{k=1}^{T}\left(\frac{g^{\left(k\right)}\left(0\right)}{k!}\right)\log_{2}\left(\frac{g^{\left(k\right)}\left(0\right)}{k!}\right)\leq 2,\\ & \omega =w_{1}+\cdots +w_{n}\leq 1,\\ & w_{i}\geq 0\;\;\forall \;i,\\ & w_{i}=w_{j}=\eta^{-1}\omega \;\;\forall \;\left\{\left(i,j\right):I\left(w_{i}\right)=I\left(w_{j}\right)=1\right\}, \end{array}$$

for Uniform(*T*) as the target distribution, and for the *k*th-derivative at x=0 of probability generating function

(30) $$g\left(x;w_{1},\ldots ,w_{n}\right)=\frac{1}{T}\sum_{j=1}^{T}x^{\left\{k\;:\;\left(I\left(w_{1}\right)r_{1j},\ldots ,I\left(w_{n}\right)r_{nj}\right)=u_{k}\right\}}.$$

While the Kelly criterion places the entire wager on the game (or games) with the greatest estimated win probability, DEPO diversifies the portfolio by distributing the percent allocation across multiple wagers according to the appropriate risk profile. For week 1, DEPO selects data point D = (1.5049, 0.0226), with estimated relative entropy of 1.5049 and expected growth rate of 0.0226. This corresponds to the optimal portfolio of three game wagers that are listed below in Table 8.

Looking at the portfolio efficiency via the risk-adjusted GROUND ratio, the DEPO portfolio has a GROUND ratio of $\Gamma_{m}=\left(0.0226-0.0135\right)/1.5049=0.6\%$, twice as efficient as the Kelly criterion portfolio at $\Gamma_{m}=\left(0.029-0.0135\right)/5.089=0.3\%$.

The actual results that follow for week 1 have KC winning by 14 (covered the spread), BAL winning by 49 (covered the spread), and LAC winning by 6 (push against the spread). Therefore, the Kelly criterion strategy experiences a gain of 20% of bankroll in week 1, while the competing DEPO strategy gains 11.78%.

#### 5.2.3. Comparison to the Kelly Criterion over Time

**Figure 5:**
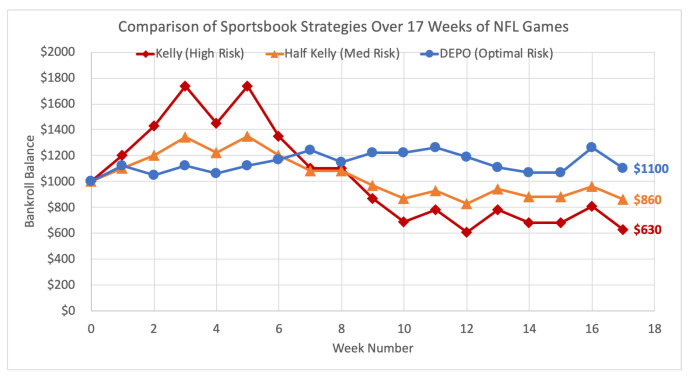
Comparison of sportsbook strategies over 17 weeks of 2019–20 season NFL games.

Demonstrated here is the performance of DEPO versus the Kelly and Kelly-variant strategies over the full 2019-20 NFL regular season. Methods in the previous Section 5.2.2 are repeated week-by-week over the course of 17 weeks. The Kelly criterion strategy wagers the optimal bet size each week on the game (or games) that have the greatest estimated probability of success. The half Kelly is the same strategy, but utilizes the fractional Kelly-variant by wagering half the Kelly criterion bet size on the same games. DEPO optimal risk strategy uses the DEPO algorithm each week to select the portfolio with the greatest expected growth rate subject to the main constraint that the portfolio has an estimated relative entropy of no greater than 2. Each strategy begins the season with $1000 and the total results are shown below in Figure 5.

Though off to a slow start, DEPO ultimately outperforms both the Kelly and half Kelly methods over the 17 week period, and it is the only strategy to produce a profit at the end. In fact, the DEPO strategy remains profitable throughout the entire experiment. As the top market consensus predictions deteriorated mid-season, the Kelly strategies suffer massive losses, while the diversification strategy of DEPO holds strong. The main purpose of DEPO is to mitigate the risk of inaccurate predictions, and once again the goal is well accomplished. In the end, DEPO finishes the season with a profit of $100, up 10% to $1100, while the half Kelly finishes at a loss of 14% and the full Kelly ends the season down 37% to $630.

## 6. Conclusions

Presented here is a new entropy-based combinatorial approach to binary option portfolio selection, called discrete entropic portfolio optimization (DEPO). DEPO introduces a robust method for evaluating the risk of binary option portfolios and gambling portfolios alike, and gives the mathematical tools to make data-driven portfolio selection decisions to mitigate risk. DEPO is robust, non-parametric, and indifferent to non-normality, asymmetry, and small sample size data, making it an ideal approach to the binary option portfolio selection problem. Compared to previous research in this space, DEPO is first to introduce the concept of managing the risk of binary options as an additional dimension to the optimization of binary option portfolios. We show how relative entropy qualifies as a convex risk measure and is therefore an ideal minimization objective for the discrete return portfolio selection problem. DEPO also adapts the Kelly criterion to a collection of binary options by extending the results to multiple wagers. By minimizing the relative entropy of portfolio returns, DEPO is able to balance risk and reward to obtain the optimal portfolio growth rate according to investor risk criteria. DEPO consistently outperforms leading Kelly criterion strategies choosing optimal portfolios of FOREX binary options. Applied to an NFL sportsbook portfolio, DEPO ultimately outperforms the industry standard quantitative methods for bet size allocation. Other possible applications of DEPO include optimizing portfolios of digital options and fixed-return options, as well as other more alternative portfolios, like sportsbooks with parlays or fantasy sports teams. Even further, any contracts with deterministic outcomes, such as Arrow–Debreu securities or related contracts, could be evaluated by their relative entropy risk and potential expected growth rate. DEPO can usher in a new range of portfolio optimization applications that were previously unavailable with the traditional mean-variance optimization or Kelly criterion alone.

## 7. Materials and Methods

FOREX binary option historical prices and outcomes sourced from the North American Derivatives Exchange website at https://www.nadex.com/market-data.

Market consensus projections sourced from www.covers.com/sports/nfl/matchups. Historical data sourced from: www.teamrankings.com/nfl under the betting view records, with past years sourced from web archives library at www.archive.org. For example, web.archive.org/web/20180319032508/www.teamrankings.com/nfl/team/baltimore-ravens.

Data and R code (R version 3.5.1) used for the portfolio selection example demonstrated in this paper can be accessed from the following DropBox sharing links,

FOREX Data: https://www.dropbox.com/s/nd6lowuz5ngpjuf/FOREX2020.csv?dl=0

FOREX Code: https://www.dropbox.com/s/1v51xhako1jqkjh/FOREX2020-DEPO.R?dl=0

NFL Data: https://www.dropbox.com/s/nd6lowuz5ngpjuf/NFL2018.csv?dl=0

NFL Code: https://www.dropbox.com/s/1v51xhako1jqkjh/NFL2018-DEPO.R?dl=0

## Figures and Tables

**Figure 4:**
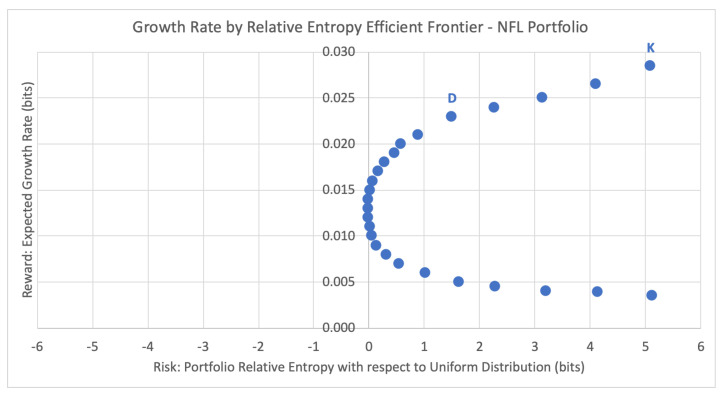
Growth rate by relative entropy efficient frontier for NFL sportsbook portfolio.

**Table 1:** Mean, in-the-money rate and estimated relative entropy (in bits) of FOREX binary options from January 2019 to January 2020.

Currency Pair	Short Name	Mean Outcome	% In-the-Money	Relative Entropy
Australian Dollar/Japanese Yen	AUD/JPY	−0.42591	0.287045	0.135127
Australian Dollar/US Dollar	AUD/USD	−0.868988	0.065506	0.651076
Euro/Pound Sterling	EUR/GBP	0.0174	0.5087	0.000218
Euro/Japanese Yen	EUR/JPY	−0.243882	0.378059	0.04334
Euro/US Dollar	EUR/USD	−0.109232	0.445384	0.008624
Pound Sterling/Japanese Yen	GBP/JPY	−0.069511	0.465244	0.003488
Pound Sterling/US Dollar	GBP/USD	−0.500692	0.249654	0.18927
US Dollar/Canadian Dollar	USD/CAD	0.468873	0.734437	0.164974
US Dollar/Swiss Franc	USD/CHF	−0.889943	0.055028	0.692615
US Dollar/Japanese Yen	USD/JPY	−0.370885	0.314557	0.101636

**Table 2:** Select FOREX binary options for 2 February 2020 11:00 p.m., with their respective contract strike price, market consensus edge, and estimated probability in-the-money.

Currency Pair	Contract Strike Price	Market Consensus Edge	*P*(In-the-Money)
Australian Dollar/Japanese Yen	AUD/JPY > 72.60	1.75%	48.25%
Australian Dollar/US Dollar	AUD/USD > 0.6700	2.25%	52.25%
Euro/Pound Sterling	EUR/GBP > 0.8420	0%	50%
Euro/Japanese Yen	EUR/JPY > 120.20	0%	50%
Euro/US Dollar	EUR/USD > 1.1080	1.5%	51.5%
Pound Sterling/Japanese Yen	GBP/JPY > 142.80	1%	49%
Pound Sterling/US Dollar	GBP/USD > 1.3180	1.5%	51.5%
US Dollar/Canadian Dollar	USD/CAD > 1.3240	2.625%	47.375%
US Dollar/Swiss Franc	USD/CHF > 0.9640	2.5%	47.5%
US Dollar/Japanese Yen	USD/JPY > 108.40	2.75%	47.25%

**Table 3:** The Kelly criterion portfolio of options for contracts expiring 2 February 2020 11:00 p.m.

Option Contract	Buy or Sell	Probability of Success	Kelly Wager %
USD/JPY > 108.40	Sell	52.75%	5.5%

**Table 4:** Discrete entropic portfolio optimization (DEPO) portfolio of options for contracts expiring 2 February 2020 11:00 p.m.

Option Contract	Buy or Sell	Probability of Success	DEPO Wager %
USD/JPY > 108.40	Sell	52.75%	0.7%
USD/CAD > 1.3240	Sell	52.625%	0.7%
USD/CHF > 0.9640	Sell	52.5%	0.7%
AUD/JPY > 72.60	Sell	51.75%	0.7%
EUR/USD > 1.1080	Buy	51.5%	0.7%
GBP/USD > 1.3180	Buy	51.5%	0.7%

**Table 5:** Mean, cover rate and estimated relative entropy of NFL teams for 2011–12 to 2018–19 seasons.

Team Name	Short Name	Mean Outcome	Cover Rate	Relative Entropy (bits)
Arizona Cardinals	ARI	0.056	0.528	0.002263
Atlanta Falcons	ATL	−0.079365	0.460317	0.004548
Baltimore Ravens	BAL	−0.081967	0.459016	0.004852
Buffalo Bills	BUF	−0.04	0.48	0.001154
Carolina Panthers	CAR	0.080645	0.540323	0.004697
Chicago Bears	CHI	−0.031746	0.484127	0.000727
Cincinnati Bengals	CIN	0.173554	0.586777	0.021838
Cleveland Browns	CLE	−0.121951	0.439024	0.010755
Dallas Cowboys	DAL	−0.02439	0.487805	0.00043
Denver Broncos	DEN	0	0.5	0
Detroit Lions	DET	−0.064516	0.467742	0.003004
Green Bay Packers	GB	0.072	0.536	0.003742
Houston Texans	HOU	−0.02439	0.487805	0.00043
Indianapolis Colts	IND	0.088	0.544	0.005593
Jacksonville Jaguars	JAC	−0.114754	0.442623	0.00952
Kansas City Chiefs	KC	0.095238	0.547619	0.006553
Los Angeles Chargers	LAC	−0.031746	0.484127	0.000727
Los Angeles Rams	LAR	−0.112903	0.443548	0.009215
Miami Dolphins	MIA	−0.04065	0.479675	0.001192
Minnesota Vikings	MIN	0.193548	0.596774	0.027194
New England Patriots	NE	0.2	0.6	0.02905
New Orleans Saints	NO	0.129032	0.572581	0.015254
New York Giants	NYG	−0.00813	0.495935	0.000047
New York Jets	NYJ	−0.081967	0.459016	0.004852
Oakland Raiders	OAK	−0.064516	0.467742	0.003004
Philadelphia Eagles	PHI	−0.055118	0.472441	0.002192
Pittsburgh Steelers	PIT	0.024	0.512	0.000415
Seattle Seahawks	SEA	0.163934	0.581967	0.019473
San Francisco 49ers	SF	0.008	0.504	0.000046
Tampa Bay Buccaneers	TB	−0.096774	0.451613	0.006767
Tennessee Titans	TEN	−0.196721	0.401639	0.028099
Washington Redskins	WAS	−0.015625	0.492188	0.000176

**Table 6:** Scheduled NFL games for Sunday 8 September 2019, with their respective Las Vegas point spreads and market consensus estimated probabilities of covering.

Away Consensus	Away Team		Home Team	Home Consensus
70%	Kansas City Chiefs −3.5	@	Jacksonville Jaguars	30%
44%	Tennessee Titans	@	Cleveland Browns −5.5	56%
53%	Atlanta Falcons	@	Minnesota Vikings −3.5	47%
43%	Washington Redskins	@	Philadelphia Eagles −10.5	57%
67%	Baltimore Ravens −7.0	@	Miami Dolphins	33%
63%	Los Angeles Rams −1.5	@	Carolina Panthers	37%
46%	Buffalo Bills	@	New York Jets −2.5	54%
44%	Cincinnati Bengals	@	Seattle Seahawks −9.5	56%
34%	Indianapolis Colts	@	Los Angeles Chargers −6.0	66%
43%	San Francisco 49ers	@	Tampa Bay Buccaneers −1.0	57%
61%	Detroit Lions −2.5	@	Arizona Cardinals	39%
58%	New York Giants	@	Dallas Cowboys −7.0	42%
51%	Pittsburgh Steelers	@	New England Patriots −5.5	49%

**Table 7:** The Kelly criterion portfolio of wagers with percent allocation for NFL 2019-20 season week 1.

Bet to Cover	Bet to Not Cover	Probability of Success	Kelly Wager %
Kansas City Chiefs −3.5	Jacksonville Jaguars	60%	20%

**Table 8:** DEPO portfolio of wagers with percent allocation for NFL 2019-20 season week 1.

Bet to Cover	Bet to Not Cover	Probability of Success	DEPO Wager %
Kansas City Chiefs −3.5	Jacksonville Jaguars	60%	5.89%
Baltimore Ravens −7.0	Miami Dolphins	58.5%	5.89%
Los Angeles Chargers −6.0	Indianapolis Colts	58%	5.89%

## Abbreviations

The following abbreviations are used in this manuscript:

AUD	Australian dollar
BAL	Baltimore Ravens
CAD	Canadian dollar
CHF	Confoederatio Helvetica (Swiss) franc
DEPO	Discrete entropic portfolio optimization
EUR	Euro
FOREX	Foreign Exchange
FRO	Fixed-return option
GBP	Great British pound (sterling)
GROUND	Growth rate over uniform divergence
IND	Indianapolis Colts
JAC	Jacksonville Jaguars
JPY	Japanese yen
KC	Kansas City Chiefs
KL	Kullback-Leibler
LAC	Los Angeles Chargers
LV	Las Vegas
MIA	Miami Dolphins
NADEX	North American Derivative Exchange
NFL	National Football League
REPO	Return-entropy portfolio optimization
USD	US dollar
